# Visinets: A Web-Based Pathway Modeling and Dynamic Visualization Tool

**DOI:** 10.1371/journal.pone.0123773

**Published:** 2015-05-28

**Authors:** Jozef Spychala, Pawel Spychala, Shawn Gomez, Gabriel E. Weinreb

**Affiliations:** 1 Visinets, Inc., Chapel Hill, North Carolina, United States of America; 2 Joint Department of Biomedical Engineering at UNC-Chapel Hill and NC State University, Chapel Hill, North Carolina, United States of America; Texas A&M University, UNITED STATES

## Abstract

In this report we describe a novel graphically oriented method for pathway modeling and a software package that allows for both modeling and visualization of biological networks in a user-friendly format. The Visinets mathematical approach is based on causal mapping (CMAP) that has been fully integrated with graphical interface. Such integration allows for fully graphical and interactive process of modeling, from building the network to simulation of the finished model. To test the performance of Visinets software we have applied it to: a) create executable EGFR-MAPK pathway model using an intuitive graphical way of modeling based on biological data, and b) translate existing ordinary differential equation (ODE) based insulin signaling model into CMAP formalism and compare the results. Our testing fully confirmed the potential of the CMAP method for broad application for pathway modeling and visualization and, additionally, showed significant advantage in computational efficiency. Furthermore, we showed that Visinets web-based graphical platform, along with standardized method of pathway analysis, may offer a novel and attractive alternative for dynamic simulation in real time for broader use in biomedical research. Since Visinets uses graphical elements with mathematical formulas hidden from the users, we believe that this tool may be particularly suited for those who are new to pathway modeling and without the in-depth modeling skills often required when using other software packages.

## Introduction

One of the persistent challenges in today’s biomedical research is the development of easy to use modeling software tools for biological networks, which would be widely accessible for bench scientists. There are a number of advanced software packages for pathway display, analysis and simulation, including: Cytoscape, Ingenuity, CellDesigner, KEGG, SymBiology in Matlab, just to name a few. These tools are primarily used by trained modelers and are not suited for biologists who do not have rigorous training in mathematics and computation, thus leaving the most numerous group of scientists without adequate computational biology tools to aid their bench work. Many of these tools also lack features that would enable interactive and dynamic ways for pathway visualization and simulation. Others employ Ordinary Differential Equations (ODE) as their mathematical method of modeling. This approach is more complex to use and larger ODE models (above 130 nodes) are more difficult to build and manage, and may also require dedicated computational facilities. These shortcomings further underscore the need for the development of novel and more accessible tools for bench scientists. Such tools, if available, would greatly accelerate the discovery process by: i) introducing bench scientists to modeling of biological systems in an interactive graphical way; ii) allowing for hypothesis testing at the early stage in the laboratory; iii) providing dynamic visualization of data in the context of signaling pathways; iv) enabling visualization of perturbations within the pathways by observing changes in pathway graphical display in an interactive and dynamic way; v) facilitating *ad-hoc* preliminary modeling of alternative network circuitry and thus determination whether further experimentation or more rigorous modeling is needed. We postulate that there is a broad and still unmet need to process, analyze/model and interpret biological information at the early stage of data acquisition and analysis. Combined with a user-friendly format that is accessible to an average health professional and scientist not trained in mathematics/statistics and programming, such tools may lower the barrier to entry for researchers who can be empowered to analyze their conceptual models and thus provide a potential boost for discovery process in biomedicine.

In this report we describe our attempt to create such a software tool based on a standardized approach using causal mapping (CMAP). In this approach, first introduced in 2006 [1], all elemental influences assumed for each biochemical reaction, in essence directly corresponding to ODE expressions, are graphically accounted for. Thus, the user may create a graphical representation of the biological network at high level of detail, apply parameters and visually monitor the network simulations in an interactive and dynamical manner. To test the performance of Visinets graphical approach we have; a) built the “*de novo*” model of EGFR and Erk1/2 signaling with manual parameter adjustment and; b) translated existing model of insulin signaling in diabetes from published ODE model [2] into CMAP formalism.

## Materials and Methods

### Graphical representation of CMAP

**Fig 1:**
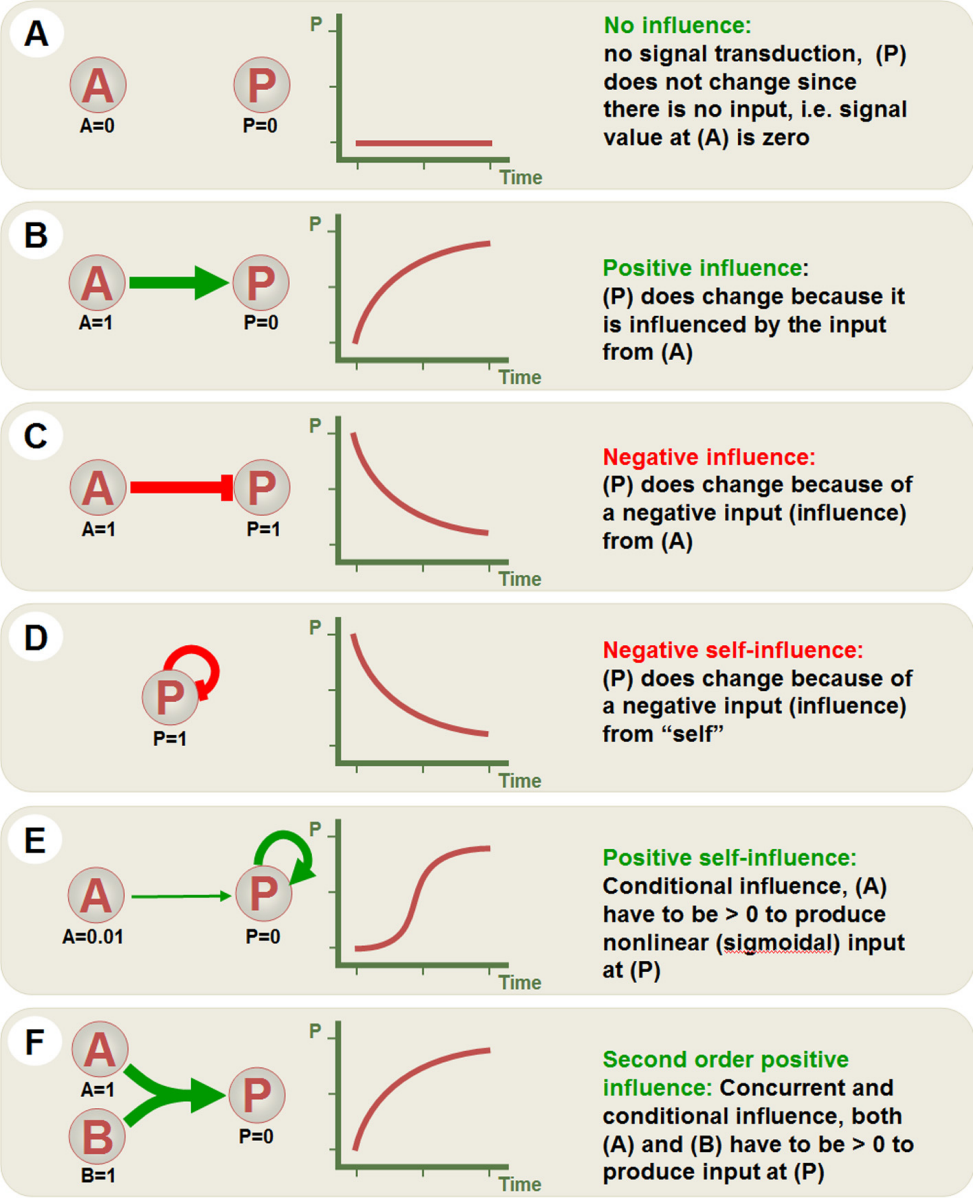
Elemental influences in Visinets. To achieve shown behavior in Visinets, all initial values for species A and P were set as shown and the weights for all influences (arrows) were set at 0.5, except for positive self-influence (E) where the influence of A on P was set at 0.01.

**Fig 2:**
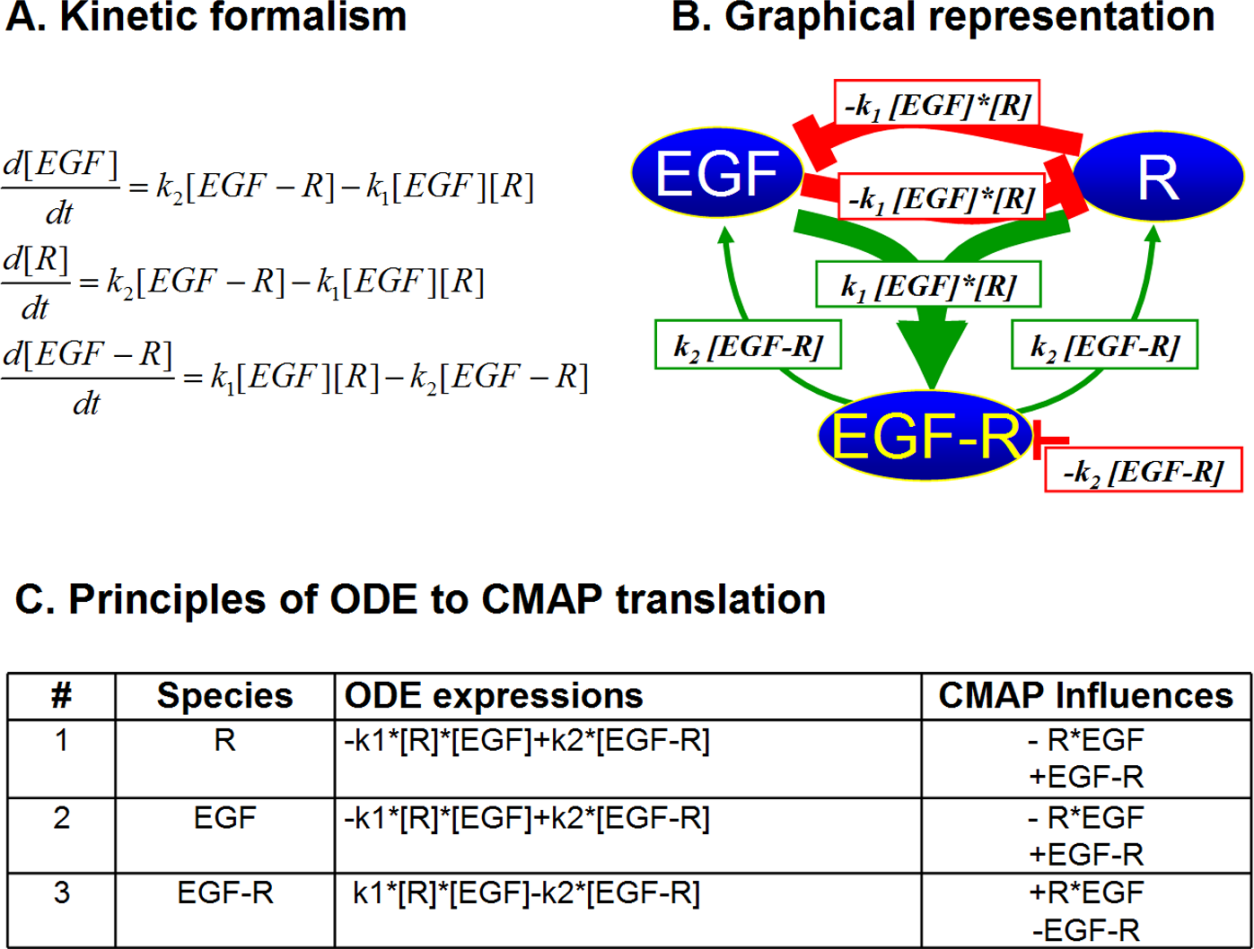
Comparative representation of binding reaction, represented here by EGFR-EGF interaction, using ODE and CMAP formalism. Translation scheme of a chemical kinetics formalism based on ordinary differential equations (A) into a CMAP representation (B), with a full one-to-one translation protocol of all ODE terms into influences (C). The reaction rate constants (k) from ODE are represented in CMAP by weights. CMAP representation allows for second order influences (e.g., k1*[R]*[EGF]) to reflect multiple causal origin of a concept’s change.

**Fig 3:**
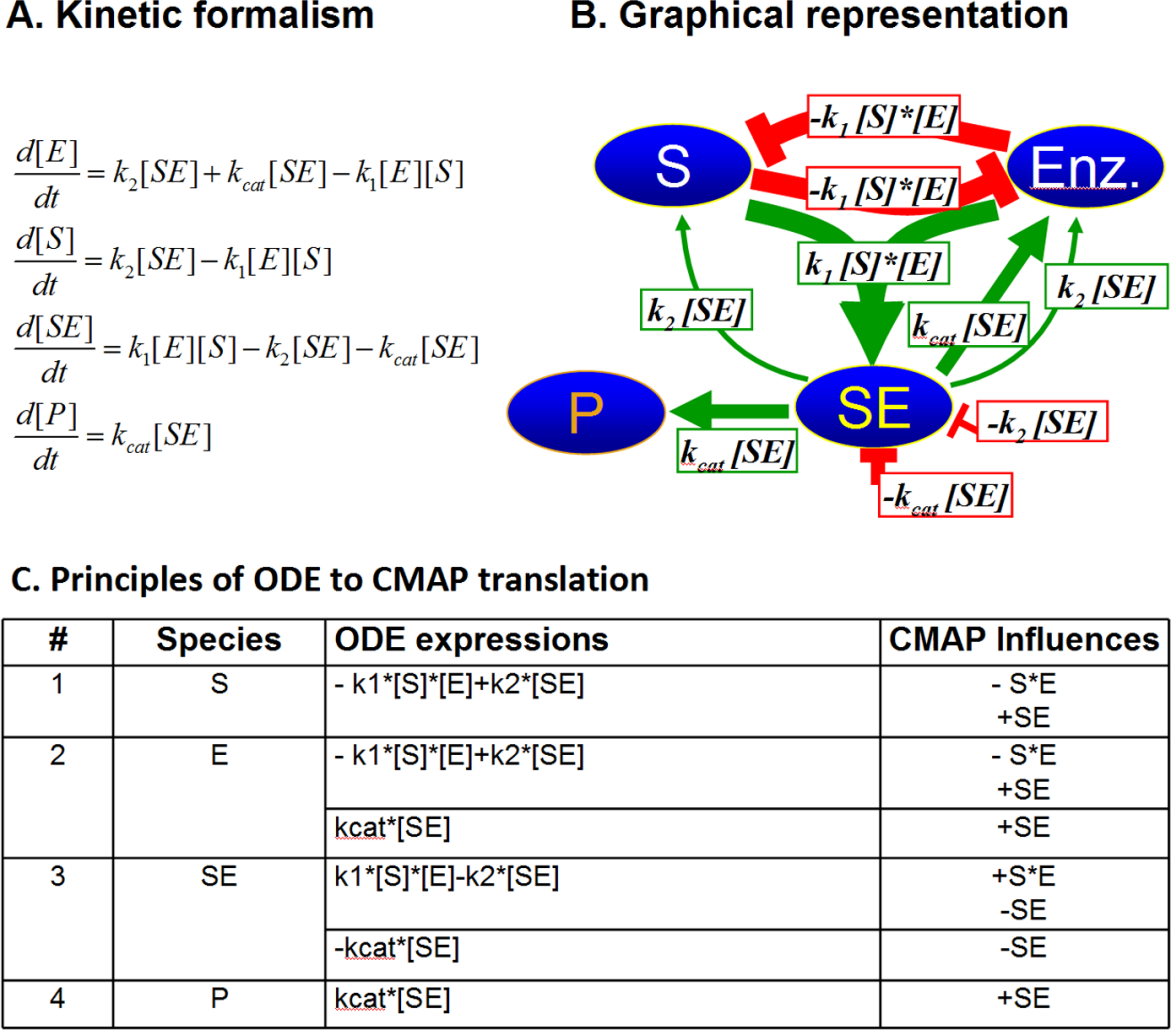
Graphical principles of conversion of an enzymatic reaction from a chemical kinetics formalism based on ordinary differential equations (A) into a CMAP representation (B), with full one-to-one translation scheme of all ODE terms into influences (C).

The basic idea of the causal mapping is to graphically represent all the components of the system of interest, along with all interactions between them, with an underlying aim to replace mathematical descriptions with graphical equivalents. While it requires simplification and generalization of the mathematical framework, it provides the power of graphical user-computer interaction particularly for users without rigorous training in mathematics of physics and chemistry. Fig 1 illustrates the basic principles of the causal mapping and their graphical depiction. In the context of biochemical reactions that take place in signaling pathways, these principles are translated into graphical representation of protein-protein interactions (for example receptor-ligand interaction) and/or enzymatic reactions (for example protein phosphorylation) shown in Fig 2 and Fig 3.

### Analytical description of CMAP

A causal map is a graph in which the components (concepts) are the nodes (species) and causal influences are the edges. The behavior of each species Cj(t) is described by the following basic CMAP equations for a network of N nodes:

$$C_{j}(t)=C_{j}(t-1)+\Lambda_{j}(C_{j}(t-1),f_{j})*f_{j}(W_{ij},C_{i}(t-1))$$ (1A)

$$\begin{array}{l} f_{j}\equiv f(x_{j})=\frac{1-e^{-\alpha_{j}x_{j}}}{1+e^{-\alpha_{j}x_{j}}},\;x_{j}=\sum_{i=1}^{N}C_{i}W_{ij}+\sum_{i=1}^{N}\sum_{k=1}^{N}C_{i}C_{k}W_{ikj}+\ldots +\sum_{i=1}^{N}\ldots \sum_{l=1}^{N}C_{i}\ldots C_{l}W_{i\ldots lj};\\ \Lambda_{j}(C_{j}(t),f_{j})=\left\{ \begin{array}{l} C_{j}^{\text{max}}-C_{j}(t),\;if\;f_{j}>0,\\ C_{j}(t),\;if\;f_{j}\leq 0, \end{array}\right. \end{array}$$ (1B)

**Fig 4:**
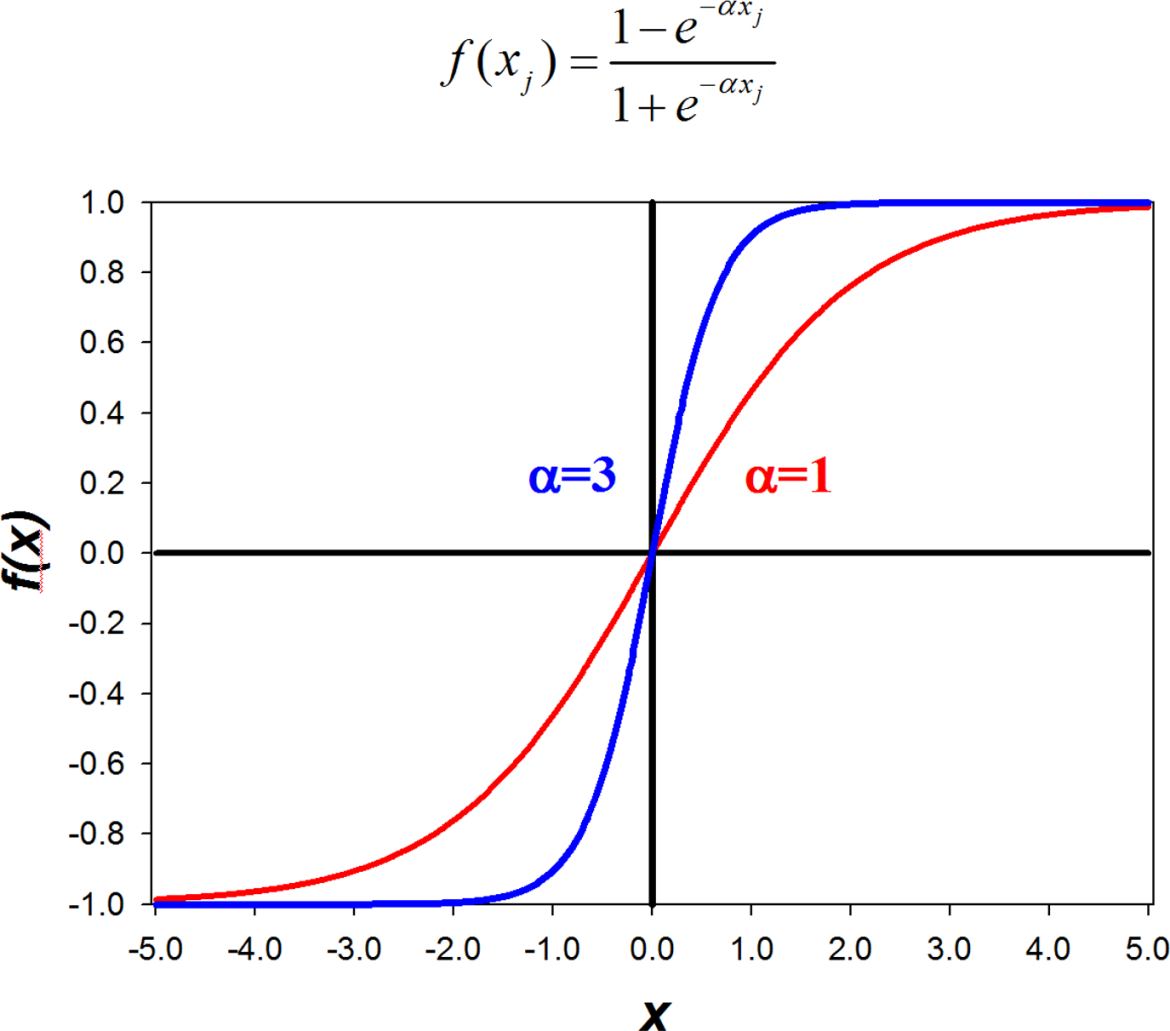
Causal function. **It describes causal interactions through concepts and weights.** The steepness of the function is determined by parameter **α**: the larger the value of **α**, the more responsive *f(x)* is to the input.

The species (***C***_***j***_ ***(t***)) can assume any value between 0 and C_j_^max^ (≤1) and are variables; they could represent species concentration and/or combination of concentration and activity. The weights (***W***_***ij***_, strengths of influences reflecting the ODE rate constants, e.g. k_1_, k_2_, and k_cat_) are constant during simulations. The absolute values of the weights are also normalized and limited to the same interval (0,1). Each influence may be assigned positive or negative value (activation or inhibition) and carry positive or negative numerical value depicted in green or red, respectively (Fig 2 and Fig 3). Multiple inputs are added and/or subtracted, depending on their values. Most species have their initial (***C***_***j***_ ***(t***)) value assigned zero, e.g. they are not active unless activated by another species (cause). The right side of **Eq (1A)** contains two factors. **Ʌ**_**j**_ restricts the species to the 0–1 scaled interval. The causal function ***f***_***j***_ includes all influences from the system (including self-influence, red line attached to one species only), as depicted in Fig 2 and Fig 3, on a given species ***C***_***j***_ ***(t)***. Parameter **α** in **Eq (1B)** (Fig 4) determines the sensitivity of a response of a species to a given input. In other words, **α** may be used, within the suggested range between 0.5 to 5 (default is set at 1.2), to increase the species responsiveness to change in input.

### Software

The front-end (GUI) and back-end of the Visinets software was developed using the Google Development Kit and Dart. Visinets uses Google's Big Table database to store all of the data that the software will create including: experimental data, models, graph visualizations, user information and analytics, references and other data. Visinets also employs Google's App Engine to host the application and APIs through multiple RESTful APIs that allow the front-end client to access, modify, and search data on the database. In addition, we use Amazon's Elastic Compute Cloud (ec2) (written in C/C++) to power the simulations. The web-based implementation ensures platform-free execution throughout all the available devices, from PC to tablets and smart phones.

### Visinets graphical user interface

Visinets graphical interface employs HTML5 technology to ensure cross-platform compatibility and takes advantage of its features: the Canvas to draw hardware accelerated pathway maps and graphs; application cache and local storage to use the software when not connected to the internet; and file-reader and drag and drop API's to efficiently use local files. Users may use Visinets in two modes: a) Build/Modify mode, where the user can construct the pathway using graphical means and manually apply initial values for parameters, and perform simulations; and b) Parameter Search mode, where user can perform global parameter search using user-selected rules.

In **Build/Modify** mode, users can take full advantage of the graphical environment and draw all components of the biological network in a causally linked manner, using both positive and negative influences (arrows/connectors). Three or four species that form a specific reaction module may be used to build signaling pathway in a more automated way. Larger network blocks, representing multiple biochemical reactions, can be selected, moved around and/or copied and pasted into new pathway files. Left side pull-out menus provide the list of all species and influences and pop-up menus for each species and influence allowing to visually inspect and edit parameters, initial conditions and graphical appearance. Several graphical features have been developed for easier workflow and more attractive visualization of signal transduction throughout the network; a) clone function to allow multiple copies of the same species to be associated with different parts of the network and thus avoiding the dense network of intersecting connections throughout the working area; b) dynamic changing of the thickness of the influence connector (arrow) in proportion to the corresponding signal intensity; and c) dynamic changing of the transparency of species that is inversely proportional to the C value (activity/concentration). Each network can be shown in **Basic View** with essential connections only showing the causality and direction of signal transduction, which is recommended for simulations and visualization and in **Full View** with all influences shown, recommended for model building, editing and/or parameter adjustments. (Supporting Information: S1 Visinets Software: Visinets software is freely available to general public as an online resource at http://www.visinets.com/index.htm with full user documentation (User Guide and Help) and a list of executable signaling pathways with embedded parameter datasets (fully user-adjustable), including those described in this report (http://www.visinets.com/pathway/list))

### Parameter Search

In Parameter Search mode the user can apply a set of rules (criteria) to search for parameters (initial conditions of species, C_ini_, and weights) automatically. The rules currently available in Visinets include Increase, Decrease, Transient up, Transient down, and Oscillations and may be set for any species. Once initiated, the search will stop at the first randomly generated set of parameters that satisfy the chosen rules and results can be viewed using the Plot function. If the result is not satisfactory the user can repeat the search for a new set of parameters or continue with manual adjustments.

## Results

To test the performance of Visinets software we have created two models of signaling pathways. First, we have re-constructed the model of EGFR signaling complex with MAPK kinase pathway and manually selected parameters to satisfy specific criteria. Second, we have translated a published ODE model of Insulin signaling using one-to-one mapping of all reactions and compared the results with the original ODE model.

### EGFR-MAPK signaling model

**Fig 5:**
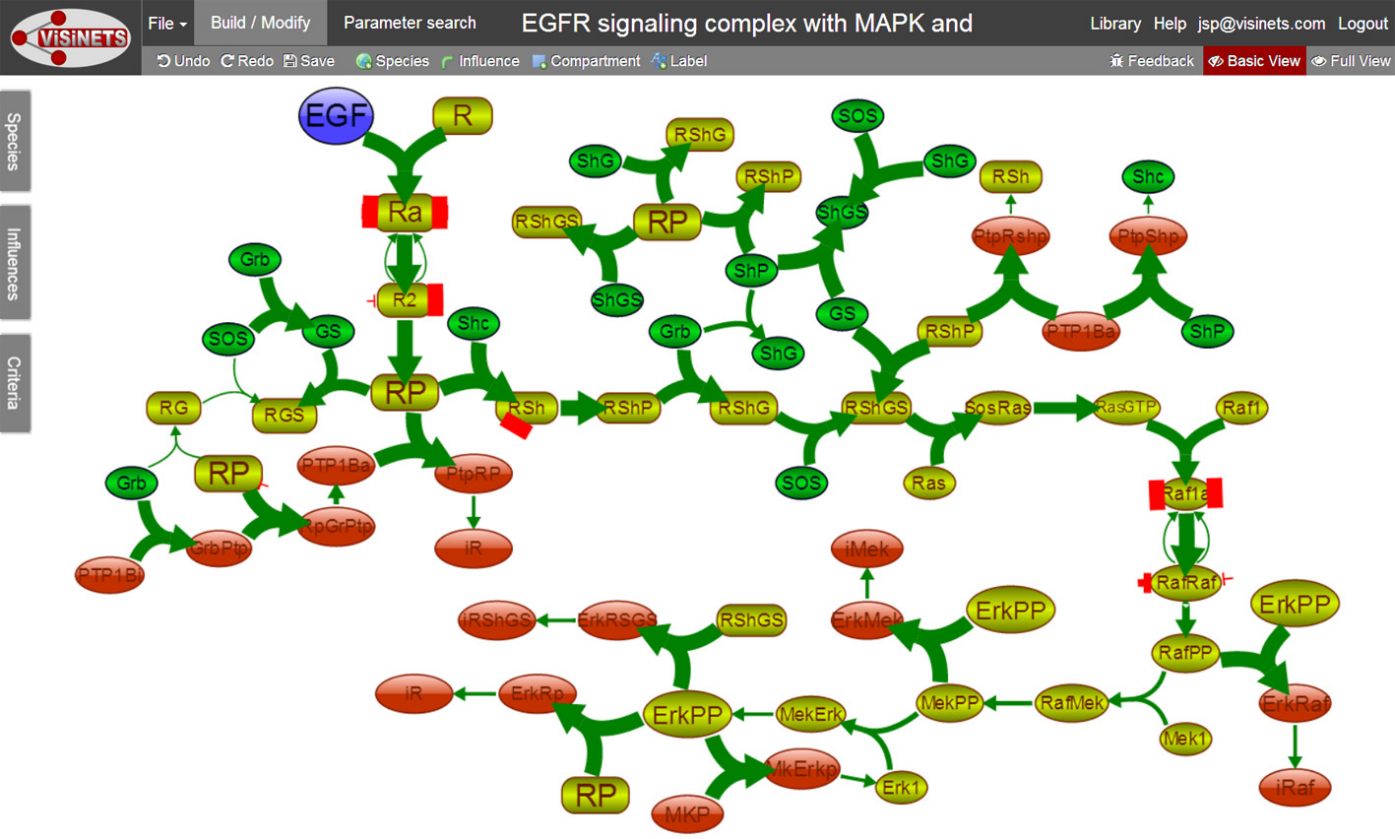
EGFR signaling pathway shown in working space of Visinets in Build/Modify mode and in default Basic View. Note the use of clones for selected species, for example for RP (phosphorylated EGFR), ErkPP (double phosphorylated Erk1/2), etc. that allow the placement of the same species in different locations, thus eliminating a dense network of crossing connections and producing a “cleaner” pathway representation. The linear “core” EGFR signaling pathway is shown in light green, adaptor proteins (grb2, Shc, SOS) and their complexes (for example GS which represents Grb2 and SOS complex) are shown in dark green, and protein phosphatases (PTP1B, activated form PTP1Ba and MKP), their inactive complexes after phosphorylation, and inactive phosphorylated Receptor, RShGS, Raf and Mek (iR, iRShGS, iRaf and iMek), are shown in red. This executable model is available with embedded parameter dataset at http://www.visinets.com/pathway/list in Featured Pathways (Supporting Information: S1 Visinets Pathway: “EGFR signaling complex with MAPK and PTP1B feedback inhibition”).

We have constructed a 70-node EGFR and MAPK signaling pathway with associated phosphoprotein phosphatases MKP and PTB1B (including 29 biochemical reactions, 229 influences and 22 clones) (Fig 5). The network of EGFR and MAPK signaling was constructed to model the behavior of combined MAPK, MKP and PTP1B feedback inhibition on EGFR signaling [3–6]. The core pathway consists of 2 initial branches of EGFR signaling, Grb2-PTP1B (phosphotyrosine phosphatase feedback) and Shc-Grb2-Sos1 (MAPK pathway). Protein adaptors Sos, Grb2 and Shc are shown within their extensive network of interactions as described in [7].

**Fig 6:**
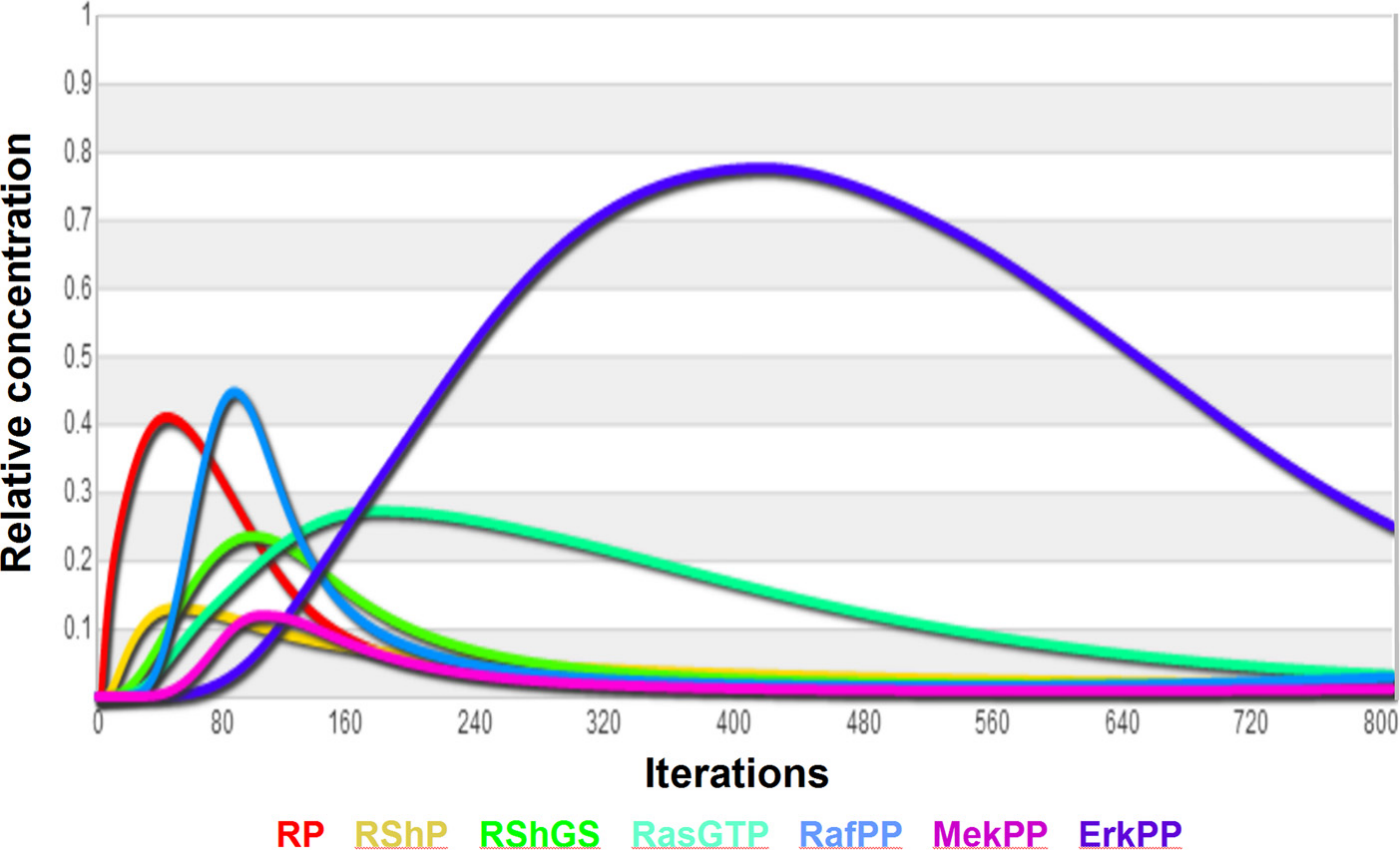
Transient activation (tyrosine or serine/threonine phosphorylation) of EGFR and MAPK pathway components shown in Visinets using Plot function. Each single iteration equates to the smallest incremental signal progression through the network (virtual time). R2—receptor dimer; Rp—phosphorylated receptor; RShP—Receptor-Shc-P; RShGS—Receptor-Shc-Grb-Sos complex; RafPP—phosphorylated raf1; MekPP—phosphorylated Mek1/2; ErkPP—phosphorylated Erk1/2.

Protein phosphatase PTP1B binds to EGFR through Grb2 adaptor protein to terminate EGFR signaling and protein phosphatase MKP2 terminates the Erk1/2 signaling. The pathway illustrates Erk1/2 feedback negative regulation of several species, including Mek1/2, Raf1 (c-Raf), Receptor-Shc-Grb2-Sos1 (RSGS) complex and tyrosine phosphorylated receptor (RP). The delayed Ser/Thr phosphorylation of EGFR by Erk1/2 remains more controversial and may be indirect. Feedback phosphorylation-inhibited species do accumulate and are named iRShGS, iR, iRaf and iMek and may be subject for dephosphorylation and recycling, or for internalization and degradation (not included in this model). Erk1/2 activity itself is terminated by protein phosphatase MKP (DUSP4). The criteria used to re-construct the core network behavior were the transient phosphorylation (activation) for RP, RSGS, Rafpp and Erkpp, generated by EGFR and several negative feedbacks of Erk1, PTP1B and MKP activities. This behavior, including relative time delay, recapitulates some of the typical regulation that is common for EGFR and MAPK pathways [3–6] (Fig 6).

The manual parameter selection strategy applied for this model involved three guiding principles, each helped by the visual analysis of signal propagation throughout the network during execution of simulation; a) slow reaction (low weight value) following fast reaction will cause the relative accumulation of the reaction intermediate; a) having only same type of inputs (“activation” or “inhibition”) can cause only corresponding monotonic change; b) to obtain “transient” (non-monotonic) response there should be at least two inputs but of different type; c) several rounds of adjustments were applied to find each weight value in the most sensitive range defined as a smallest weight adjustment made to cause the largest change in activity, and d) final execution of simulation with visual determination of node transparency (as a graphical representation of activity/concentration) to identify potential remaining signaling roadblocks or diversions. These adjustments did not need to be exhaustive, in the above case the criteria were satisfied within 2 hours of manual weight parameter manipulations. The **α** parameter was left at default value of 1.2 for all but RafPP and ErkPP, in which case it was set at 4 to enhance its responsiveness to input and induce a more pronounced”switch-like” behavior. The sum of these adjustments produced RafPP responding to EGF levels in a concentration dependent manner. However, the less responsive way of ErkPP may be due to incomplete negative regulation of activated Erk1/2 represented in our scheme.

### Insulin signaling in diabetes

**Fig 7:**
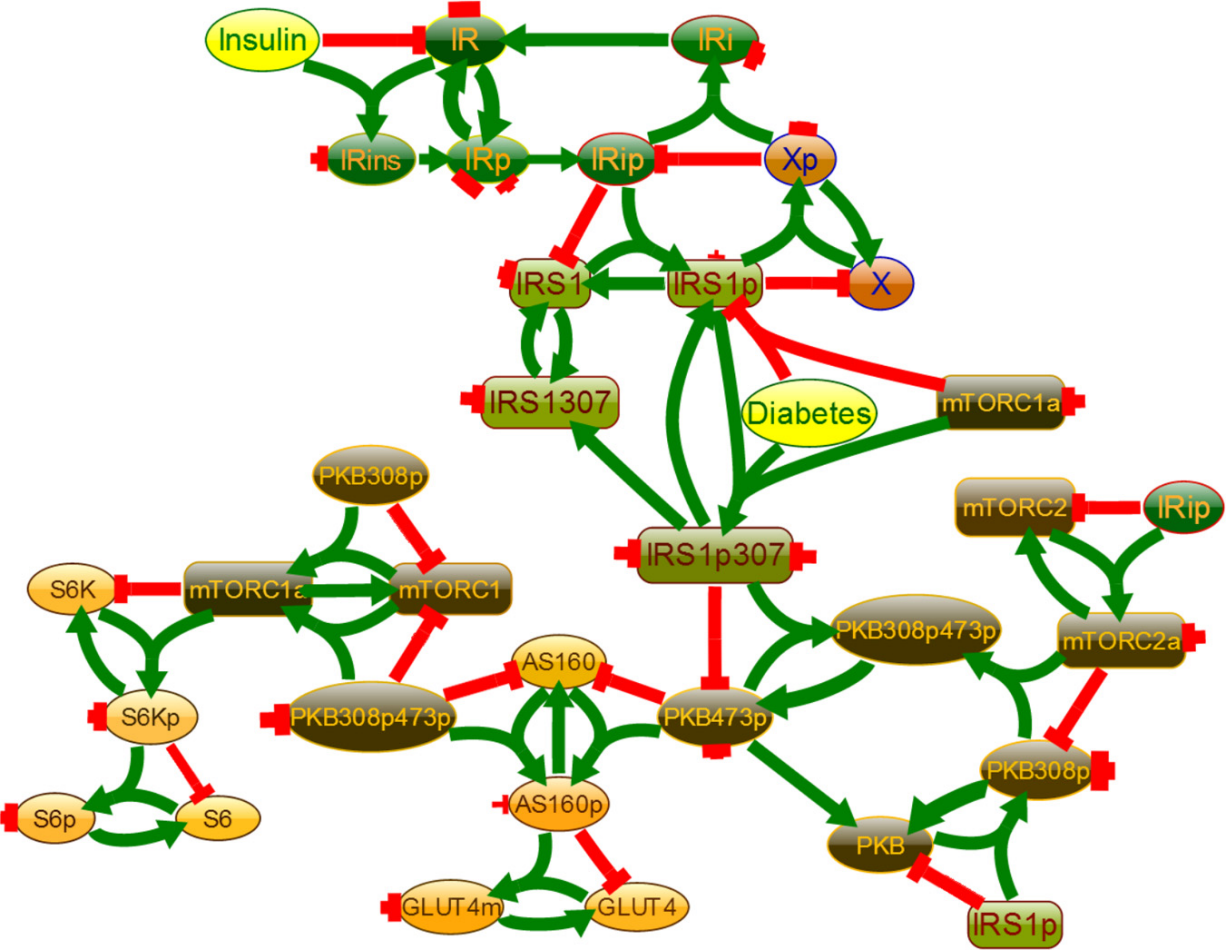
Simplified Insulin signaling pathway, reconstructed in Visinets as described in [2]. This executable model is available with embedded parameter datasets at http://www.visinets.com/pathway/list in Featured Pathways (Supporting Information: S2 Visinets Pathway: “Insulin signaling in Diabetes”).

To further test the performance of Visinets, and to directly compare our approach to ODE method, we used an existing ODE model of insulin signaling in normal and type 2 diabetes (T2D) cells [2] and translated it, ODE expression-to-CMAP influence (one-to-one), using the general scheme described above in Fig 2 and Fig 3. The Table 1 lists all the corresponding reactions we used to re-create the model in Visinets. Since the original model has been intentionally reduced to 27 species and deviates somewhat from strict mass-action formalism, the resulting CMAP representation is simplified accordingly. The re-created insulin signaling pathway is shown in Fig 7.

**Table 1:** The list of all reactions and species, as shown for the simplified insulin signaling model [2] and their equivalent CMAP influences.

#	Component (derivative)	ODE Expressions	CMAP influences	Initial values
**1**	**IR**	IRp * k1g	+ IRp	1
		+ IRi * k1r	+ IRi	
		- IR * k1a * insulin	- IR * insulin	
		- k1basal * IR	- IR	
**2**	**IRp**	k1basal * IR	+ IR	0
		+ IRins * k1c	+ IRins	
		- IRp * k1d	- IRp	
		- IRp * k1g	- IRp	
**3**	**IRins**	IR * k1a * insulin	+ IR * insulin	0
		- IRins * k1c	- IRins	
**4**	**IRip**	IRp * k1d	+ IRp	0
		- IRip * k1f * Xp	- IRip* Xp	
**5**	**IRi**	IRip * k1f * Xp	+ IRip* Xp	0
		- IRi * k1r	- IRi	
**6**	**IRS1**	IRS1p * k2b	+ IRS1p	0.8
		+ IRS1307 * k2g	+ IRS1307	
		- IRS1 * k2a * IRip	- IRS1* IRip	
		- IRS1 * k2basal	- IRS1	
**7**	**IRS1p**	IRS1 * k2a * IRip	+ IRS1* IRip	0
		+ IRS1p307 * k2d	+ IRS1p307	
		- IRS1p * k2b	- IRS1p	
		-IRS1p*k2c*mTORC1a*diabetes	- IRS1p*mTORC1a*diabetes	
**8**	**IRS1p307**	IRS1p*k2c*mTORC1a*diabetes	+IRS1p*mTORC1a*diabetes	0
		- IRS1p307 * k2d	- IRS1p307	
		- IRS1p307 * k2f	- IRS1p307	
**9**	**IRS1307**	IRS1p307 * k2f	+ IRS1p307	0.2
		+ IRS1 * k2basal	+ IRS1	
		- IRS1307 * k2g	- IRS1307	
**10**	**X**	Xp * k3b	+ Xp	1
		- X * k3a * IRS1p	- X* IRS1p	
**11**	**Xp**	X * k3a * IRS1p	+ X * IRS1p	0
		- Xp * k3b	- Xp	
**12**	**PKB**	k4b * PKB308p	+ PKB308p	0.7
		+ k4h * PKB473p	+ PKB473p	
		- k4a * PKB* IRS1p	- PKB* IRS1p	
**13**	**PKB308p**	k4a * PKB* IRS1p	+ PKB* IRS1p	0
		- k4b * PKB308p	- PKB308p	
		- k4c * PKB308p * mTORC2a	- PKB308p * mTORC2a	
**14**	**PKB473p**	k4f * PKB308p473p	+ PKB308p473p	0
		- k4e * PKB473p * IRS1p307	- PKB473p * IRS1p307	
		- k4h * PKB473p	- PKB473p	
**15**	**PKB308p473p**	k4c * PKB308p * mTORC2a	+ PKB308p * mTORC2a	0
		+ k4e * PKB473p * IRS1p307	+ PKB473p * IRS1p307	
		- k4f * PKB308p473p	- PKB308p473p	
**16**	**mTORC1**	mTORC1a * k5b	+ mTORC1a	0.9
		- mTORC1 * k5a1 * PKB308p473p	- mTORC1* PKB308p473p	
		- mTORC1 * k5a2 * PKB308p	- mTORC1 * PKB308p	
**17**	**mTORC1a**	mTORC1 * k5a1 * PKB308p473p	+ mTORC1 * PKB308p473p	0
		+ mTORC1 * k5a2 * PKB308p	+ mTORC1* PKB308p	
		- mTORC1a * k5b	- mTORC1a	
**18**	**mTORC2**	k5d * mTORC2a	+ mTORC2a	0.9
		- mTORC2 * k5c * IRip	- mTORC2 * IRip	
**19**	**mTORC2a**	mTORC2 * k5c * IRip	+ mTORC2* IRip	0
		- k5d * mTORC2a	- mTORC2a	
**20**	**AS160**	AS160p * k6b	+ AS160p	0.7
		- AS160* k6f1 * PKB308p473p	- AS160* PKB308p473p	
		- AS160*k6f2 * PKB473p^n6^ /(km6n6 + PKB473p^n6^)	- AS160* PKB473p	
**21**	**AS160p**	AS160* k6f1 * PKB308p473p	+ AS160* PKB308p473p	0
		+ AS160* k6f2 * PKB473p^n6^ /(km6n6 + PKB473p^n6^)	+ AS160* PKB473p	
		- AS160p * k6b	- AS160p	
**22**	**GLUT4m**	GLUT4 * k7f * AS160p	+ GLUT4* AS160p	0
		- GLUT4m * k7b	- GLUT4m	
**23**	**GLUT4**	GLUT4m * k7b	+ GLUT4m	0.6
		- GLUT4 * k7f * AS160p	- GLUT4* AS160p	
**24**	**S6K**	S6Kp * k9b1	+ S6Kp	0.9
		- S6K * k9f1 * mTORC1a^n9^/(km9^n9^ + mTORC1a^n9^)	- S6K * mTORC1a	
**25**	**S6Kp**	S6K * k9f1 * mTORC1a^n9^ /(km9^n9^ + mTORC1a^n9^)	+ S6K * mTORC1a	0
		- S6Kp * k9b1	- S6Kp	
**26**	**S6**	S6p * k9b2	+ S6p	0.9
		- S6 * k9f2 * S6Kp	- S6 * S6Kp	
**27**	**S6p**	S6 * k9f2 * S6Kp	+ S6 * S6Kp	0
		- S6p * k9b2	- S6p	
**28**	**I (insulin)**			0
**29**	**diabetes**			1

### Parameter search for insulin signaling model

**Fig 8:**
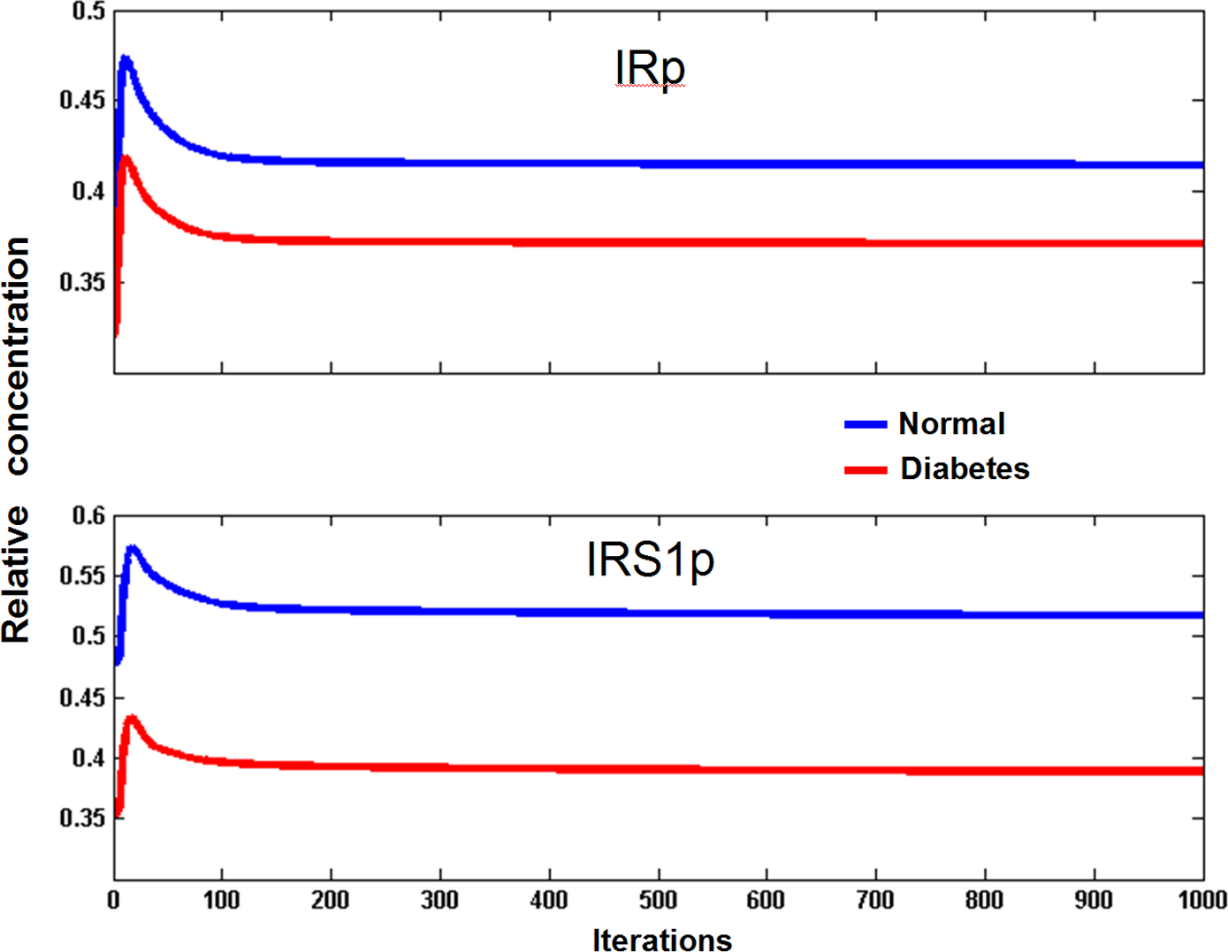
Transient activation (tyrosine phosphorylation) of IR and IRS1. The phosphorylated species shown are the sum of multiple forms of both IR and IRS1.

To obtain parameter sets for the simulation of Insulin pathway in normal and pathological states and to compare the ODE and CMAP simulations directly, we exported CMAP code from Visinets and run parameter search and simulations in Matlab (MathWorks, Inc.). The transient phosphorylation of Insulin Receptor and IRS1 were used as search rules and the search was conducted in a step-wise manner:

1. Export the model from Visinets (File/Export Model) to create a programing code for further simulations.
2. Generate the 1 million random sets of weights (corresponding to rate constants in the ODE model) so that max(W)/min(W) = 1000 and α-s (0.5–3) and the values were evenly distributed in these ranges.
3. Run simulation for each weight set until steady state was achieved with the initial values for the species shown in the Table 1 for both normal and T2D cells. The differences between the two types of cells were (in correspondence with Figure 1 in [2]):

IR(T2D) = 0.55* IR;

GLUT4(T2D) = 0.5* GLUT4;

Diabetes (T2D) = 0.15* Diabetes;

1. After achieving the steady states values for all species, we used those as initial conditions to run the simulation in the presence of insulin (insulin = 1) and tested for two rules: transient response for measuredIRp = IRp+IRip and measuredIRS1p = IRS1p + IRS1p307 for both normal and T2D conditions. Representative simulations qualitatively confirming the finding from the original ODE model are shown in Fig 8.

1. Using obtained parameters sets that satisfy the above rules, we found those that belong to both normal and T2D ensembles and saved them for further analysis.
2. We ran these sets to satisfy observations presented in Fig 5B from [2]: the responses curves from normal cells lie higher than the ones for the T2D cells. The satisfaction of these two rules was the final point of our selection process.

It is also important to note that to achieve pathway behavior as described in the original paper [2], the Visinets user may also follow the guidelines described for the EGFR-MAPK pathway described above, and manually find the parameter set that satisfies the original criteria.

### Computational efficiency

We have compared computational efficiency of simulations with CMAP and ODE approaches by running simulations of 3 different biological pathways: Insulin signaling in the Normal state as described above, EGFR signaling pathway as described by Kholodenko & co-authors [4], and EGFR signaling as described by Borisov & co-authors [8]. The comparison was performed using build-in feature of Matlab. The computational efficiency, as defined by time per iteration point and calculated as a ratio of ODE simulation time to CMAP simulation time, varied from 4.29 for Insulin signaling model, to 9.13 for EGFR model [8], and to 64.25 for EGFR model [4]. This comparison shows a significant advantage of CMAP models when compared head to head in computational efficiency.

## Discussion

The underlying mathematical approach in Visinets (Causal Mapping (CMAP)) is a modeling method that allows for the integration of diverse data into a single model of networks organized in causally linked relationships. Initially applied to model of cortical contraction [1, 9], CMAP is a semi-quantitative approach based on a detailed description of all network interactions and is particularly useful when detailed knowledge of those interactions is absent and specific parameter values need to be estimated. CMAP approach lies between coarse-grained (e.g. Boolean and Petri nets) methods, and highly detailed mechanistic (e.g. ODE based) methods (Table 2). As with ODEs, CMAP allows for continuous modeling with standardized kinetic equations for each species and thus exhibiting significant advantage over the simpler discrete Boolean and Rule Based methods which can’t be parameterized and thus describe the model behavior with significantly lower level of detail [10].

**Table 2:** Causal mapping represents an approach with intermediate level of detail relative to that required for differential equations and Boolean network approaches.

Comparison of CMAP with other approaches		
Highly Coarse Grained	Detailed Coarse Grained	Highly Detailed
Boolean; Petri nets	Causal mapping (CMAP)	**Differential Equations:** Chemical kinetics; Mechanics; Stochastic physics

CMAP permits the investigation of system’s dynamics, with all its elements connected in a biologically and causally meaningful way (as with differential equation approaches) and is tolerant to knowledge gaps, discrepancies and uncertainties. Since this standardized method for pathway modeling can easily be executed within the user-friendly graphical interface, it allows for an intuitive and non-technical network analysis for bench biologists, geneticists and health professionals. Although other standardized mathematical approaches have been proposed [10, 11], they were not developed into fully user-friendly and graphically oriented software packages. There are few software packages available that are user-friendly (e.g., Cell Collective [12] or RuleBender [13]) but they are using other modeling methods such as rule-based modeling and/or Boolean algebra and thus having their own limitations. Other known tools such as Cytoscape or Ingenuity Pathway Analysis are very useful for data mining, pathway graphical illustration, however they lack the power of simulation and dynamic visualization.

To evaluate the “ease of use” and simulation performance, and potentially broader user appeal in biomedical research, we have successfully re-created the behavior of EGFR and MAPK pathway by employing manual selection of weight parameter for each reaction. Such method may also be used for refining pathway behavior after initial automated parameter search. Though in some cases the manual method may seem tedious, the process of weight (i.e., reaction rate constants) adjustments itself may provide the user with an intuitive way for better understanding of pathway dynamics, relationships between different sections of the networks, and mechanistic relationships such as positive and negative feedback loops. In fact, this more intuitive manual way of model refinement and associated benefit of learning pathway dynamics may better help the user to discern between similar network topologies and settings and find the most optimal network circuitry for further experimentation and analysis. As such Visinet’s dynamic computational models can be considered a means of conceptual model verification, by which models generated by researchers from the understanding of their research field can be verified computationally and the outcomes simulated, and thus the behavioral consequences of the researcher’s proposed topology/hypothesis can be evaluated in an easy to follow graphical way [14]. This way of using Visinets could also serve as potentially great training/educational tool. Visinets free online access offers users several additional signaling pathways to further illustrate the potential of the software and its research application for the broader community of biomedical bench scientists.

The direct comparison of insulin ODE and CMAP models provides strong evidence that both approaches give very similar outcomes. Furthermore, direct comparison of computational efficiency for 3 independent models show the clear advantage for CMAP approach for use in simulations of biological pathways on desktop computers. The insulin signaling pathway, studied in more detail above, is also an example of using an existing ODE model and importing it into Visinets. If such model is available, the translation into Visinets may be relatively straightforward and allows the user to generate simulations and dynamic pathway illustrations within the extensive graphical capabilities of Visinets. Furthermore, user may import and then modify, add or delete species (nodes) or influences (edges) or even combine smaller models into larger one within the Visinets platform. The software module of ODE model import function into Visinets is being currently developed. In a reverse process, if necessary, the Visinets model/network can also be exported into more capable software packages where more extensive parameter search and parameter space study using qualitative rules could be performed. Importantly, Visinets will continue to extend the range of statistical tools and graphical features in Visinets modeling software, thus potentially alleviating the need for further analysis in other software packages.

Due to its graphical capabilities, the usefulness of Visinets may be extended beyond modeling of signaling pathways, and into dynamic visualization of other biological processes. Thus, the modeling capability of Visinets, in combination with attractive visualization, may offer a unique way for the integrated graphical analysis of different biological scenarios and also use it for presentation purposes.

In this version Visinets may be a particularly useful tool for semi-quantitative analysis of pathway models that are larger than 50–100 nodes, a large size considered for computationally more demanding ODE models. With the zoom function currently in place, Visinets graphical working space can practically accommodate pathway models larger than 300 nodes.

## Supporting Information

### S1 CMAP Code.

The code for Visinets CMAP modeling engine has been deposited for free use at https://github.com/paulspychala/CMAPdart under open source MIT license.

### S1 Visinets Pathway.

EGFR signaling complex with MAPK and PTP1B feedback inhibition. This pathway model has parameter dataset embedded in the pathway and is listed in Featured Pathways at http://www.visinets.com/pathway/list.

### S2 Visinets Pathway.

Insulin signaling in Diabetes. This pathway model has parameter datasets embedded in the pathway and is listed in Featured Pathways at http://www.visinets.com/pathway/list.

### S1 Visinets Software.

Visinets software is freely available to general public as an online resource at http://www.visinets.com/index.htm with full user documentation (User Guide and Help) and a list of executable signaling pathways with embedded parameter datasets (fully user-adjustable), including those described in this report (http://www.visinets.com/pathway/list).
